# Signal Transducer and Activator of Transcription 4 (STAT4) Association with Pituitary Adenoma

**DOI:** 10.3390/medicina60111871

**Published:** 2024-11-14

**Authors:** Greta Gedvilaite-Vaicechauskiene, Loresa Kriauciuniene, Rasa Liutkeviciene

**Affiliations:** Laboratory of Ophthalmology, Neuroscience Institute, Medical Academy, Lithuanian University of Health Sciences, Eiveniu 2, LT-50161 Kaunas, Lithuania; loresa.kriauciuniene@lsmuni.lt (L.K.); rasa.liutkeviciene@lsmuni.lt (R.L.)

**Keywords:** pituitary adenoma, STAT4, rs10181656, rs7574865, rs7601754, rs10168266

## Abstract

*Background/Objectives:* This study aims to investigate whether Signal Transducer and Activator of Transcription 4 (STAT4) influences the anti-tumor immune response and is possibly involved in the initiation or relapse of pituitary adenomas (PAs) by examining *STAT4* polymorphisms and serum levels. This research seeks to uncover potential connections that could inform future therapeutic strategies and improve our understanding of PA pathogenesis. *Materials and Methods:* This study was conducted at the Laboratory of Ophthalmology, Lithuanian University of Health Sciences. DNA was extracted from peripheral venous blood samples, and the genotyping of four STAT4 SNPs (rs7574865, rs10181656, rs7601754, and rs10168266) was performed using real-time PCR with TaqMan^®^ Genotyping assays. The serum STAT4 levels were measured via ELISA, and the optical density was read at 450 nm. Genotype frequencies, allele distributions, and serum STAT4 levels were statistically analyzed to assess associations with pituitary adenoma occurrence. *Results:* A binary logistic regression revealed that the *STAT4* rs7574865 GT + GG genotypes vs. TT were associated with 1.7-fold increased odds of PA occurrence under the dominant genetic model (*p* = 0.012). The stratification by gender showed no significant associations in females; however, in males, the *STAT4* rs10168266 CC + CT genotypes compared to TT were linked to 2.5-fold increased odds of PA under the dominant genetic model (*p* = 0.005). *STAT4* rs10181656, rs7574865, rs7601754, and rs10168266 were analyzed to evaluate the associations with the pituitary adenoma size. We found that the *STAT4* rs7574865 GG genotype was statistically significantly less frequent in the macro PA group compared to in the reference group (*p* = 0.012). For PA relapse, the rs7574865 G allele was less frequent in the PA group without relapse (*p* = 0.012), and the GT + GG genotypes were associated with a 1.8-fold increase in the PA group without relapse occurrence (*p* = 0.008). The serum STAT4 levels were higher in the PA patients compared to those of the reference group (*p* < 0.001). Elevated STAT4 serum levels were observed in PA patients with the STAT4 rs10181656 CC or CG genotypes (CC: *p* = 0.004; CG: *p* = 0.023), and with the rs7574865 GG or GT genotypes (GG: *p* = 0.003; GT: *p* = 0.021). The PA patients with the *STAT4* rs7601754 AA genotype exhibited higher serum levels compared to those of the reference group (*p* < 0.001). Similarly, higher serum levels were found in the PA patients with the *STAT4* rs10168266 CC or CT genotypes (CC: *p* = 0.004; CT: *p* = 0.027). A haplotype frequency analysis revealed no statistically significant results. *Conclusions:* The *STAT4* genotypes were significantly associated with the PA occurrence, size, and relapse. Elevated serum STAT4 levels were observed in the PA patients, highlighting its potential role in PA pathogenesis.

## 1. Introduction

STAT4 (Signal Transducer and Activator of Transcription 4) is a protein that plays an important role in the regulation of immune reactions and various genes involved in the immune system [1]. Activated by the JAK-STAT pathway, STAT4 regulates immune reactions and inflammation [2]. Chronic inflammation is a known risk factor for the development of cancer. Increased levels of inflammation can create a microenvironment that promotes tumor growth and progression [3]. Therefore, in cases where STAT4 is dysregulated or overactive, it may contribute to an inflammatory state that may influence oncogenesis.

STAT4 plays an important role in regulating gene expression and modulating the immune system’s response to various signals [4]. It belongs to the STAT family of proteins, which are involved in signal transduction and transcriptional activation in response to cytokines and growth factors [5]. Different types of cytokines can activate STAT4 in different cells, such as tumor or immune cells, via the JAK-STAT pathway [4].

**Figure 1:**
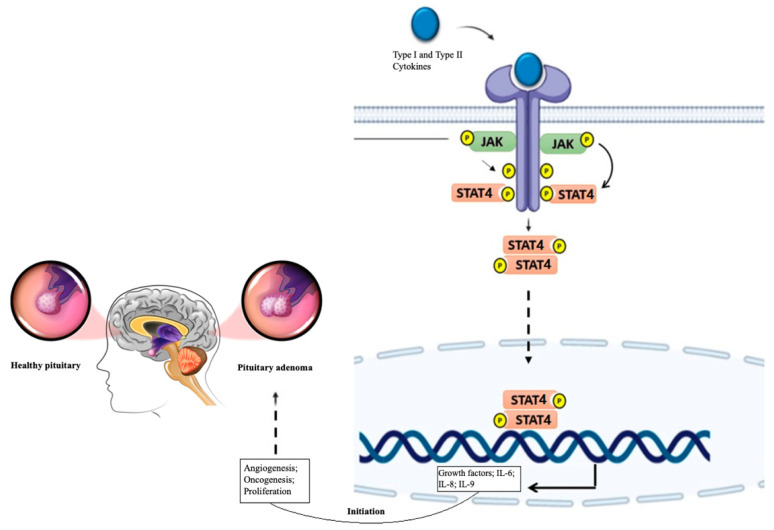
The JAK-STAT4 signaling pathway involves JAK proteins, which remain inactive near the intracellular domains of receptors in the absence of cytokines. When a cytokine binds to its receptor, JAK proteins and the receptor’s intracellular domains become phosphorylated. This activation recruits and phosphorylates STAT4 protein, causing them to dimerize and translocate to the nucleus, where they initiate the transcription of genes involved in cell proliferation. JAK: Janus kinase protein; STAT: signal transducers and activators of transcription.

The JAK-STAT signaling pathway is essential for immune system regulation and cell processes like division, differentiation, and death. Dysregulation of this pathway contributes to tumorigenesis [6]. In the absence of cytokines, JAK proteins remain inactive near the intracellular domains of receptors. When a cytokine binds to its receptor, JAK proteins and the receptor’s intracellular domains become phosphorylated. This activation recruits and phosphorylates STAT4 proteins, causing them to dimerize and translocate to the nucleus, where they initiate the transcription of genes involved in cell proliferation [7,8]. Signaling by type I and II cytokine receptors is crucial in this process. Upon cytokine binding to the extracellular domain of these receptors, JAKs are activated, which in turn phosphorylate multiple substrates, including STAT4. The phosphorylated STAT4 then dimerizes, translocates to the nucleus, binds DNA, and regulates gene expression, promoting various cellular processes such as proliferation, angiogenesis, or oncogenesis (Figure 1) [9,10].

In vivo and in vitro studies over the last few decades have shown that STAT4 can induce inflammation, inhibit tumor growth or promote tumor development by regulating many aspects of the immune response [11]. In addition, STAT4 regulates tumor cell migration and proliferation [4]. Since it can be activated in both tumor and immune cells, it is suspected that STAT4 may modulate the interaction between tumor cells and host immunity [12].

Pituitary adenomas (PAs) are mostly benign, but they display a wide range of behaviors and health impacts [13,14]. Research indicates that the pathogenesis of PA may be associated with gene mutations, chromosomal abnormalities, DNA methylation, microRNA regulation, and transcription factor modulation [15,16]. The abnormal expression of cell cycle genes, activation of oncoproteins, or loss of suppressor factors in the pituitary can lead to disrupted growth factor signaling. Understanding these subcellular mechanisms is key to developing markers for tumor aggression and new targeted therapies [17].

To date, there is no known direct link between STAT4 and the development of PAs. Thus, in the present study, we aim to investigate whether STAT4 influences the anti-tumor immune response and is possibly involved in the initiation or relapse of PAs by examining *STAT4* polymorphisms and serum levels. This research seeks to uncover potential connections that could inform future therapeutic strategies and improve our understanding of PA pathogenesis.

## 2. Methods

This study was conducted in the Laboratory of Ophthalmology, Lithuanian University of Health Sciences. Kaunas Regional Biomedical Research Ethics Committee approved the study (Approval number: BE-2-47, dated 25 December 2016). All participants were introduced to the structure and objectives of the present study before its execution. An Informed Consent Form was obtained from all subjects involved in the study.

### 2.1. DNA Extraction and Genotyping

The DNA was extracted from peripheral venous blood samples (leucocytes) collected in 200 µL tubes using a genomic DNA extraction kit utilizing silica-based membrane technology (GeneJET Genomic DNA Purification Kit, Thermo Fisher Scientific, Vilnius, Lithuania) based on the manufacturer’s recommendations. The study analyzed four single nucleotide polymorphisms (SNPs) within the *STAT4* gene:

- rs7574865: this SNP involves a G>T substitution located in intron 3 at chromosome position 191,964,633, denoted as NC_000002.12:191099907: T>G in HGVS nomenclature.
- rs10181656: a C>G substitution located in intron 3 at chromosome position 191,969,879, denoted as NC_000002.12:191105152: C>G in HGVS nomenclature.
- rs7601754: a G>A substitution located in intron 4 at chromosome position 191,940,045, denoted as NC_000002.12:191075724: G>A in HGVS nomenclature.
- rs10168266: a C>T substitution located in intron 5 at chromosome position 191,935,804, denoted as NC_000002.12:191071077: C>T in HGVS nomenclature.

Single nucleotide polymorphisms of *STAT4* were detected using the real-time polymerase chain reaction (RT-PCR) method. TaqMan^®^ Genotyping assays were used to determine SNPs according to the manufacturer’s protocols by a StepOne Plus (Applied Biosystems, Waltham, MA, USA). A 5% subset of samples underwent repetitive analysis for all three SNPs to ensure accuracy, confirming consistent genotyping results between the initial and repetitive assessments.

### 2.2. Serum Level Measurements

Serum levels of STAT4 were measured twice in both control subjects and patients with PA. This determination was conducted through an enzyme-linked immunosorbent assay (ELISA) employing the Signal Transducer And Activator Of Transcription 4 (STAT4) ELISA kit (Cat. No. abx156860), with a standard curve sensibility range of 0.112–20 ng/mL and a sensitivity of <0.12 ng/mL. The analysis of serum levels followed the manufacturer’s guidelines using a Multiskan FC Microplate Photometer (Thermo Scientific, Waltham, MA, USA) at a wavelength of 450 nm. The optical density (OD) at 450 nm, recorded using a microplate reader, facilitates accurate concentration calculations, particularly within blood serum. This process entails a standardized method of measurement and calculation, utilizing reference standard readings to estimate concentrations from the generated standard STAT4 curve.

### 2.3. Study Group

The study included 496 subjects, divided into a reference group (*n* = 357) and a pituitary adenoma (PA) group (*n* = 139). The reference group was matched to the PA group for gender and age (*p* = 0.550 and *p* = 0.763, respectively). Detailed demographic information for all subjects is provided in Table 1.

Patients diagnosed with pituitary adenoma (PA) were recruited from a specialized endocrinology center. Healthy controls were recruited from the general population through advertisements and health check-up camps, ensuring a similar age and gender distribution as the PA group.

Inclusion criteria for the PA group included the following:

- Diagnosed and confirmed PA through magnetic resonance imaging (MRI).
- Good general health.
- Informed consent.
- Age 18 years and above.
- No other tumors.

The control group included participants who matched the PA group in gender and age distribution, had no history of pituitary adenoma or other tumors, were in good general health, provided informed consent, and were aged 18 years and above.

Exclusion criteria for both groups included the presence of other tumors or severe comorbidities affecting study outcomes.

Blood samples were collected from PA patients after their initial diagnosis during their first clinic visit. For the control group, samples were collected from healthy subjects meeting the inclusion criteria during general health check-ups.

### 2.4. Statistical Analysis

The demographic characteristics between the reference and pituitary adenoma (PA) groups were compared using the Pearson chi-square test, Student’s *t*-test, and Mann–Whitney U test. Genotype and allele frequencies for *STAT4* rs10181656, rs7574865, rs7601754, and rs10168266 were presented as percentages. Binary logistic regression was used to analyze the association of these SNPs with PA occurrence, estimating odds ratios (ORs) and 95% confidence intervals (CIs). The most suitable genetic model was selected based on the lowest Akaike information criterion (AIC). Logistic regression results were expressed using various genetic models: co-dominant, dominant, recessive, overdominant, and additive.

Nonparametric Mann–Whitney U tests were used for non-normally distributed data. All statistical analyses were performed with SPSS version 29.0 (Statistical Package for the Social Sciences, Chicago, IL, USA). Haplotype analysis for the PA and reference groups was conducted using SNPStats software. Linkage disequilibrium (LD) was measured and presented as D’ and r^2^ values. Haplotype associations with PA were calculated by logistic regression and reported as ORs and 95% CIs. A two-sided test with a *p*-value less than 0.05 was considered statistically significant, with Bonferroni correction applied to adjust for multiple comparisons (*p* = 0.0125 (0.05/4)).

## 3. Results

The frequencies of genotypes and alleles were analyzed within the study groups for the following SNPs: *STAT4* rs10181656, rs7574865, rs7601754, and rs10168266. We found that the rs7574865 GG genotype was less frequent in the PA than in the reference group (46.0% vs. 58.5%, *p* = 0.012). However, after applying the Bonferroni correction to adjust for multiple comparisons, no statistically significant differences were found in the distribution of the *STAT4* rs10181656, rs7601754, and rs10168266 genotypes and alleles between the patients with PA and the reference group for the selected SNPs (refer to Table 2).

The binary logistic regression revealed that the *STAT4* rs7574865 GT + GG genotype vs. TT is associated with about 1.7-fold increased odds of PA occurrence under the dominant genetic model (OR = 1.655; CI: 1.115–2.455; *p* = 0.012) (Table 3).

The frequencies of genotypes and alleles for the selected SNPs were analyzed within the study groups, stratified by gender; however, no statistically significant results were found in the females (Supplementary Material Table S1), while in the males, the *STAT4* rs10168266 CC genotype and C allele were less frequent in the PA group than in the reference group (50.9% vs. 72.1%, *p* = 0.005; 71.9% vs. 84.6%, *p* = 0.004, respectively) (Table 4).

A binary logistic regression analysis was conducted on the patients with PA and the reference group to investigate the associations of the selected SNPs with the PA occurrence by gender. The analysis did not reveal any statistically significant results when analyzing the females (Supplementary Material Table S2), while in the males, the following statistically significant results were found: the *STAT4* rs10168266 CC + CT genotypes compared to the TT genotype were associated with 2.5-fold increased odds of pituitary adenoma occurrence under the most robust dominant genetic model (OR = 2.490; CI: 1.313–4.724; *p* = 0.005) (Table 5).

The *STAT4* rs10181656, rs7574865, rs7601754, and rs10168266 single nucleotide polymorphisms were analyzed to evaluate the associations with the pituitary adenoma size. Analyzing *STAT4* rs7574865, we found that the GG genotype is statistically significantly less frequent in the macro PA group compared to in the reference group (43.8% vs. 58.5%, *p* = 0.012) (Table 6).

However, the binary logistic regression analysis results revealed no statistically significant results after the Bonferroni correction was applied (Supplementary Material Table S3).

The frequencies of the genotypes and alleles for the selected SNPs were analyzed within the PA group with or without relapse. The analysis revealed that the *STAT4* rs7574865 G allele was less frequent in the PA group without relapse than in the reference group (66.8% vs. 75.5%, *p* = 0.012) (Table 7).

The binary logistic regression analysis results revealed that the *STAT4* rs7574865 GT + GG genotypes compared to the TT genotype is associated with 1.8-fold increased odds of PA without relapse occurrence under the most robust dominant genetic model (OR = 1.803; CI: 1.166–2.788; *p* = 0.008) (Table 8).

**Figure 2:**
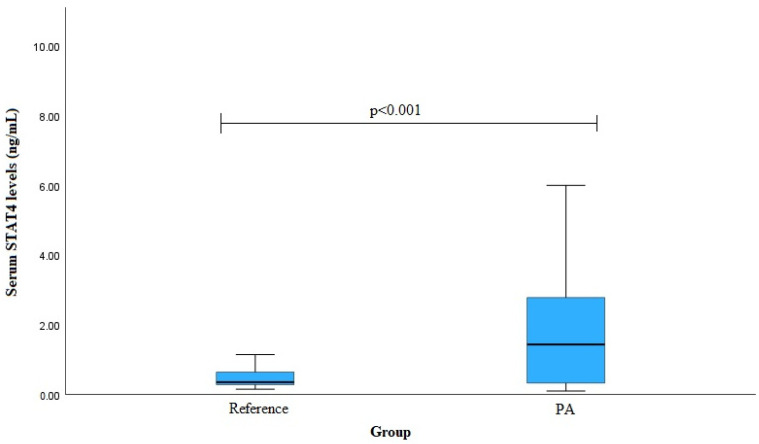
Serum STAT4 levels (ng/mL) in PA vs. reference groups. Mann–Whitney U Test was used.

The STAT4 serum levels in the PA patients and reference group subjects were evaluated. The analysis revealed that the STAT4 serum levels were elevated in the PA group compared to the reference group (median (IQR): 1.434 (2.498) ng/mL vs. 0.352 (0.382) ng/mL, *p* < 0.001) (Figure 2).

**Figure 3:**
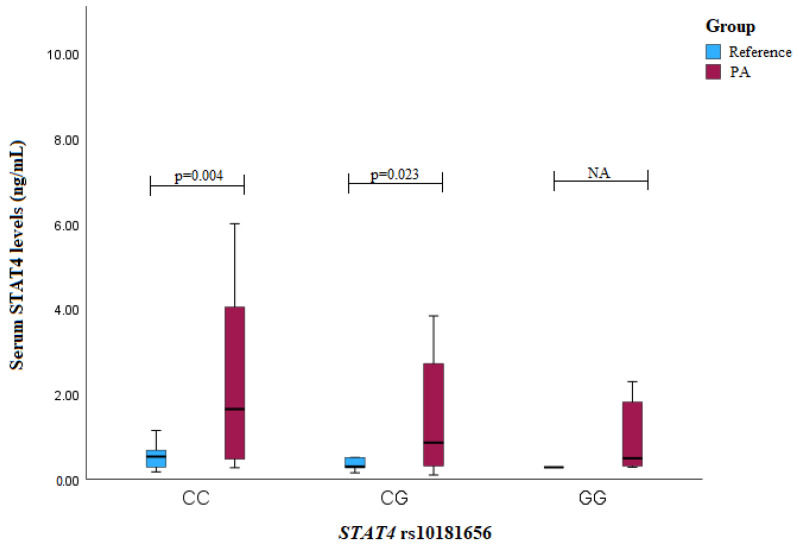
Serum STAT4 levels (ng/mL) in PA vs. reference groups compared between *STAT4* rs10181656 genotypes.

The serum STAT4 levels were compared among the different genotypes for *STAT4* rs10181656, rs7574865, rs7601754, and rs10168266. The analysis revealed that the PA patients with the *STAT4* rs10181656 CC or CG genotypes exhibited higher serum levels compared to the reference group subjects (CC genotype: median (IQR): 1.645 (3.873) vs. 0.532 (0.435), *p* = 0.004; CG genotype: median (IQR): 0.858 (2.424) vs. 0.296 (0.361), *p* = 0.023) (Figure 3).

**Figure 4:**
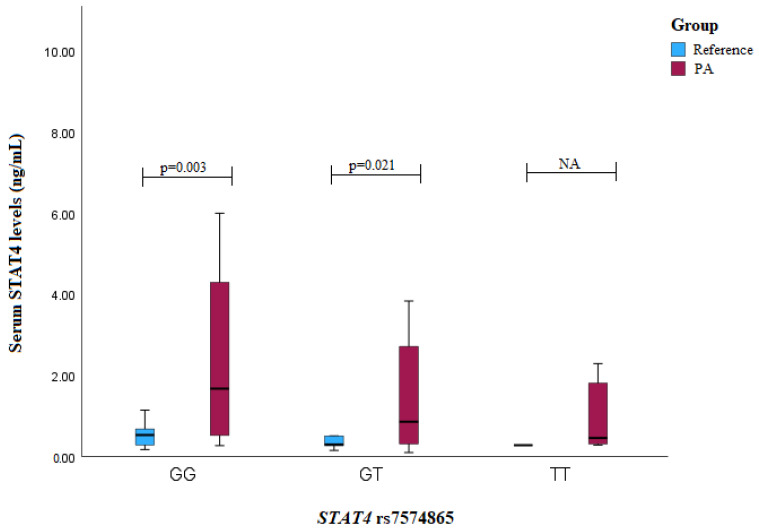
Serum STAT4 levels (ng/mL) in PA vs. reference groups compared between *STAT4* rs7574865 genotypes.

Similar results were found when analyzing the serum levels of the PA patients with *STAT4* rs7574865: the patients with the GG or GT genotypes exhibited higher serum levels compared to those of the reference group subjects (GG genotype: median (IQR): 1.675 (0.435) vs. 0.532 (0.435), *p* = 0.003; GT genotype: median (IQR): 0.858 (2.424) vs. 0.296 (0.361), *p* = 0.021) (Figure 4).

**Figure 5:**
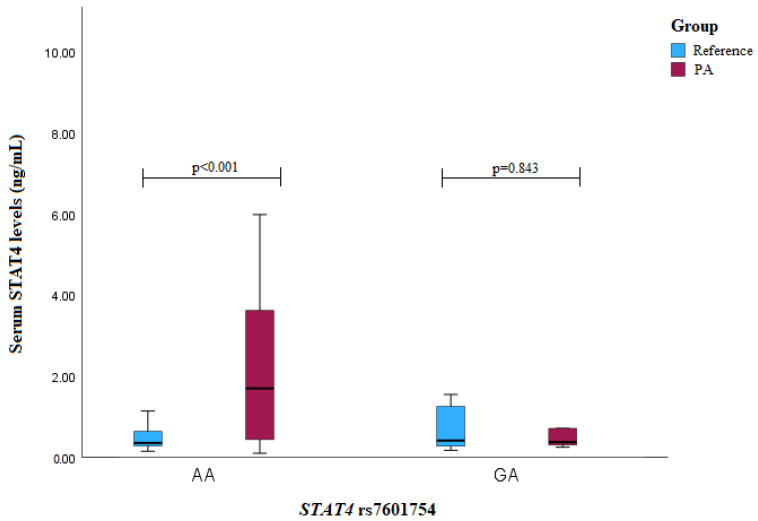
Serum STAT4 levels (ng/mL) in PA vs. reference groups compared between *STAT4* rs7601754 genotypes.

The PA patients with the *STAT4* rs7601754 AA genotype exhibited higher serum levels compared to those in the reference group subjects (median (IQR): 1.702 (3.301) vs. 0.352 (0.362), *p* < 0.001) (Figure 5).

**Figure 6:**
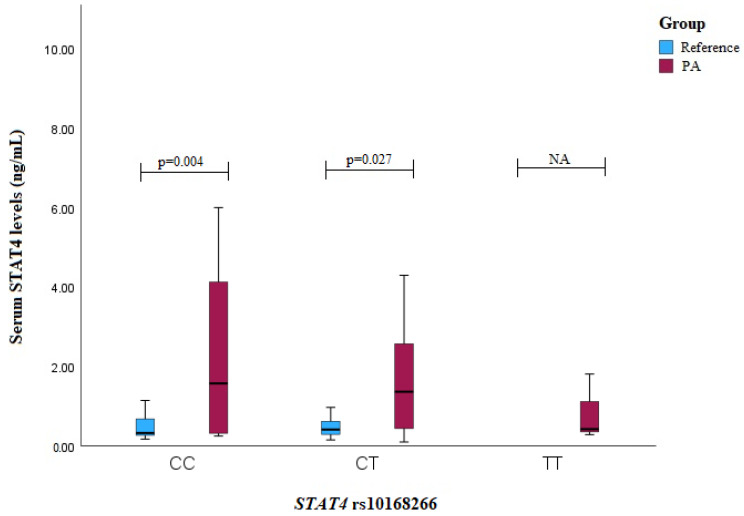
Serum STAT4 levels (ng/mL) in PA vs. reference groups compared between *STAT4* rs10168266 genotypes.

The PA patients with the STAT4 rs10168266 CC or CT genotypes exhibited higher serum levels compared to those in the reference group subjects (CC genotype: median (IQR): 1.573 (3.981) vs. 0.326 (0.459), *p* = 0.004; CT genotype: median (IQR): 1.365 (2.336) vs. 0.411 (0.345), *p* = 0.027) (Figure 6).

We performed a haplotype association analysis of *STAT4* rs10181656, rs7574865, rs7601754, and rs10168266 in the patients with PA. The pairwise linkage disequilibrium between the SNPs in the PA patients is shown in Supplementary Material Table S4.

Also, we analyzed the haplotype frequencies; however, the analysis revealed no statistically significant results (Supplementary Material Table S5).

## 4. Discussion

Pituitary adenomas (PAs) are common brain tumors that are typically slow-growing, benign, and treatable with surgery or medication. However, some PAs exhibit aggressive growth, resisting conventional treatments and leading to early relapse [18,19]. Affecting the central nervous system, PAs may cause symptoms primarily due to the compression of surrounding structures [20].

Dysregulated STAT4 can lead to chronic inflammation, creating a microenvironment that promotes tumor growth and progression [21,22]. The *STAT4* gene, located on chromosome 2q33, encodes a transcription factor essential for inflammation development in various immune-mediated diseases [23]. As SNPs are the most common genetic variants in the human genome, this makes them key targets for studying genetic associations. Understanding these variations is crucial for personalized medicine, which is the future of human well-being [24]. *STAT4* polymorphisms have been extensively studied in immune regulation disorders, including rheumatoid arthritis, polymyositis/dermatomyositis, and systemic lupus erythematosus [25,26,27]. Previous studies suggest that the role of *STAT4* polymorphisms in influencing gene expression may involve altered mRNA splicing or transcription factor binding. However, further research is needed to clarify the exact molecular mechanisms involved.

Currently, no established links exist between *STAT4* and PAs. Therefore, our study aimed to investigate whether there is an association between the *STAT4* SNPs rs10181656, rs7574865, rs7601754, and rs10168266, as well as the STAT4 levels, with the occurrence, size, and relapse of PAs.

C. Wang et al. reported that the *STAT4* rs7574865 GG genotype is a risk factor for hepatocellular carcinoma (HCC), with elevated STAT4 levels in the serum and peritumoral tissue of HCC patients with the GG genotype [28]. A meta-analysis by X. Zhao et al. suggested a reduced risk of hepatitis B virus (HBV)-induced HCC associated with the minor rs7574865 T allele in Asian populations [29], while the G allele was linked to an increased risk of HBV-induced liver cancer [30]. Y. Ma and colleagues found the minor allele T of rs7574865 might be protective against lung cancer, suggesting a similar influence on cancer occurrence [31]. Our study revealed that the *STAT4* rs7574865 GT + GG genotype is associated with 1.7-fold increased odds of PA occurrence under the dominant genetic model (*p* = 0.012). The rs7574865 GG genotype was less frequent in the macro PA group compared to the reference group (*p* = 0.012). For PA relapse, the rs7574865 G allele was less frequent in the PA group without relapse (*p* = 0.012), and the GT + GG genotypes were associated with a 1.8-fold increase in PA without relapse occurrence (*p* = 0.008). These findings suggest that the G allele might be protective against PA occurrence and recurrence. Additionally, the rs7574865 GG and GT genotypes exhibit higher serum STAT4 levels compared to those of the reference group, suggesting that this SNP may influence STAT4 expression and contribute to PA pathogenesis.

Most studies on *STAT4* rs10181656, rs7601754, and rs10168266 relate to autoimmune diseases rather than tumorigenesis.

The SNP rs10181656 showed evidence for an association with psoriatic arthritis (PsA) [32]. Another study found that PsA patients more frequently exhibited the GG genotype and G allele of rs10181656, suggesting its implication in PsA development [33]. H. S. Lee et al. found that minor alleles of rs10181656 might contribute to earlier T1D development by influencing cytokine signaling [34]. Although there are no studies linking rs10181656 with PA, our study revealed that the *STAT4* rs10181656 CC and CG genotypes are associated with elevated serum STAT4 levels in PA patients, indicating that this SNP may influence STAT4 expression and contribute to PA pathogenesis.

The SNP rs7601754 is primarily analyzed in endometriosis [35] and systemic lupus erythematosus [36]. H. Yuan et al.’s meta-analysis suggested that the T allele of *STAT4* rs7601754 might be a risk factor for SLE [36]. Additionally, rs7601754 likely represents an independent risk variant for SLE, with significant enrichment of the risk allele in European and Asian cohorts [37]. While there are no studies analyzing rs7601754 with PA, our study revealed that PA patients with the rs7601754 AA genotype exhibited higher serum levels compared to the reference group subjects. This suggests that the rs7601754 AA genotype might influence STAT4 expression, contributing to PA occurrence.

The minor allele frequencies of rs10168266 were significantly increased in primary biliary cirrhosis (PBC) patients compared to controls [38]. A meta-analysis suggested that the polymorphisms rs7574865, rs7601754, and rs10168266 in *STAT4* were significantly associated with the PBC risk [39]. *STAT4* rs10168266 was also associated with reduced breast cancer risk in females [40]. In our study, gender stratification showed no significant results in females, but in males, the *STAT4* rs10168266 CC + CT genotypes were linked to 2.5-fold increased odds of PA (OR = 2.490; CI: 1.313–4.724; *p* = 0.005). Additionally, the *STAT4* rs10168266 CC and CT genotypes are associated with elevated serum STAT4 levels in PA patients, indicating that this SNP may play a role in increasing STAT4 expression and contributing to PA development.

While only a few studies associate *STAT4* SNPs with tumorigenesis, researchers have demonstrated that STAT4 expression is related to cancer. Y. Li et al. found that ovarian cancer patients with high STAT4 expression had a worse prognosis compared to those with low STAT4 expression, noting a significant upregulation of STAT4 in cancerous tissues compared to normal ones. These findings suggest that elevated STAT4 expression may be associated with poorer outcomes in ovarian cancer [41]. Similarly, A. Li et al. found that STAT4 expression was significantly higher in acute myeloid leukemia (AML) bone marrow tissue samples compared to normal bone marrow tissue samples, indicating an upregulation of STAT4 in AML. This suggests that increased STAT4 expression may play a role in the pathogenesis of AML [42]. M. Li and colleagues discovered that in vitro experiments showed a significant upregulation of STAT4 expression in bladder cancer (BCa) cell lines compared to the human normal bladder epithelial cell line [43]. Our study revealed that the STAT4 levels were significantly higher in PA patients compared to those in the reference group (median [IQR]: 1.434 [2.498] ng/mL vs. 0.352 [0.382] ng/mL, *p* < 0.001). This suggests that STAT4 may promote tumorigenesis and could serve as an independent biomarker for predicting PA prognosis. Additionally, Y. Huang et al. investigated that prolonged IL-12 stimulation reduces the STAT4 protein levels in NK cells, suggesting that IL-12 specifically downregulates STAT4 expression, which may modulate STAT4 signaling in NK cells [44]. Furthermore, a decreased percentage and mean fluorescence intensity of Natural Killer Group 2, Member D (NKG2D)-expressing NK cells were found in patients with prolactinoma and non-secreting pituitary adenoma compared to healthy subjects, indicating that the immune escape of pituitary adenomas is related to the downregulation of NKG2D [45]. Our study observed elevated STAT4 serum levels in patients with pituitary adenomas (PAs) compared to the reference group, suggesting a potential role for *STAT4* in PA pathogenesis. This finding aligns with previous research indicating that specific single nucleotide polymorphisms (SNPs) within the *STAT4* gene may influence the STAT4 expression and contribute to disease susceptibility. For example, the T allele of rs7574865 has been associated with increased STAT4 mRNA and protein levels, potentially conferring a higher risk for autoimmune disorders [46]. Conversely, Wang et al. reported that the GG genotype of rs7574865 was linked to elevated STAT4 levels in hepatocellular carcinoma, highlighting the context-dependent effects of this SNP [28]. Additionally, studies have shown that the G allele of rs10181656 and the T allele of rs10168266 are associated with reduced serum STAT4 levels in age-related macular degeneration [47], while the rs7601754 variant has been linked to lower STAT4 levels and increased multiple sclerosis risk [48]. These findings suggest that intronic *STAT4* SNPs may modulate gene expression, potentially through mechanisms such as altered mRNA splicing or linkage disequilibrium with other causative mutations [49]. However, additional studies are needed to elucidate the exact mechanisms by which selected SNPs influence STAT4 expression in PAs and to clarify the role of STAT4 as a novel marker for PAs.

In summary, our findings suggest that specific *STAT4* polymorphisms, particularly rs7574865, are associated with the risk and progression of PA. The elevated STAT4 levels in the PA patients indicate that STAT4 may play a role in tumorigenesis, and could potentially serve as a biomarker for PA prognosis. However, further research is required to elucidate the precise mechanisms underlying these associations.

Despite the valuable insights gained from this study, several limitations should be acknowledged. Firstly, the sample size was limited to the number of available cases, which may affect the generalizability of the findings. Additionally, the study focused solely on specific SNPs in the *STAT4* gene, potentially overlooking other relevant genetic variants that could contribute to PA pathogenesis. Future research should aim to include larger, more diverse populations and explore additional genetic factors to validate and expand upon these findings.

## 5. Conclusions

*STAT4* genotypes, particularly rs7574865, were significantly associated with the occurrence, size, and relapse of PAs. Elevated serum STAT4 levels in PA patients further suggest a potential role for STAT4 in PA pathogenesis. These findings indicate that STAT4 may serve as both a biomarker for PA prognosis and a target for future therapeutic interventions. Further research is needed to elucidate the underlying mechanisms by which STAT4 influences PA development and to explore its potential as a therapeutic target.

## Figures and Tables

**Table 1:** Demographic characteristics of the study population.

Characteristics		Group		*p*-Value
		PA Group	Reference Group	
Gender	Females, *n* (%)	82 (59.0)	221 (61.9)	0.550 *
	Males, *n* (%)	57 (41.0)	136 (38.1)	
Age, mean (SD)		54.4 (20.5)	53.9 (14.0)	0.763 **
Size: Micro/Macro		50/89	Not Applicable	Not Applicable
Relapse: PA with relapse/PA without relapse		32/107	Not Applicable	Not Applicable

* Pearson’s chi-square test was used; ** Student’s *t* test was used; PA—pituitary adenoma; SD—standard deviation; *p*-value: significance level (alpha = 0.05).

**Table 2:** Genotype and allele frequencies of single nucleotide polymorphisms (*STAT4* rs10181656, rs7574865, rs7601754, and rs10168266) within PA and reference groups.

Polymorphism	PA, *n* (%)	Reference Group, *n* (%)	*p*-Value
*STAT4* rs10181656			
CC	65 (46.8)	208 (58.3)	0.067
CG	62 (44.6)	123 (34.5)	
GG	12 (8.6)	26 (7.3)	
Total	139 (100)	357 (100)	
Allele			
C	192 (69.1)	539 (75.5)	0.039
G	86 (30.9)	175 (25.5)	
*STAT4* rs7574865			
GG	64 (46.0) ^1^	209 (58.5) ^1^	0.042
GT	62 (35.0)	121 (33.9)	
TT	13 (2.5)	27 (7.6)	
Total	139 (100)	357 (100)	
Allele			
G	190 (68.3)	539 (75.5)	0.022
T	88 (31.7)	175 (25.5)	
*STAT4* rs7601754			
AA	117 (84.2)	270 (75.6)	0.118
AG	20 (14.4)	80 (22.4)	
GG	2 (1.4)	7 (2.0)	
Total	139 (100)	357 (100)	
Allele			
A	254 (91.4)	620 (86.8)	0.048
G	24 (8.6)	94 (13.2)	
*STAT4* rs10168266			
CC	81 (58.3)	238 (66.7)	0.182
CT	50 (36.0)	106 (29.7)	
TT	8 (5.8)	13 (3.6)	
Total	139 (100)	357 (100)	
Allele			
C	212 (76.3)	582 (81.5)	0.063
T	66 (23.7)	132 (18.5)	

^1^ *p* = 0.012 (GG vs. GT + TT); *p*-value—significance level. Bonferroni correction applied to the significance level when *p* < 0.0125 (0.05/4).

**Table 3:** Binary logistic regression analysis of *STAT4* rs10181656, rs7574865, rs7601754, and rs10168266 within patients with pituitary adenoma and reference group subjects.

Model	Genotype/Allele	OR (95% CI)	*p*-Value	AIC
*STAT4* rs10181656				
Co-dominant	CG vs. GG CC vs. GG	1.613 (1.613–2.438) 1.477 (0.706–3.091)	0.023 0.301	587.053
Dominant	CG + CC vs. GG	1.589 (1.072–2.357)	0.021	585.106
Recessive	CC vs. GG + CG	1.203 (0.589–2.456)	0.612	560.184
Overdominant	CG vs. CC + GG	1.532 (1.027–2.284)	0.036	586.085
Additive	G	1.365 (1.009–1.846)	0.044	586.409
*STAT4* rs7574865				
Co-dominant	GT vs. TT GG vs. TT	1.673 (1.105–2.534) 1.572 (0.767–3.225)	0.015 0.217	586.111
Dominant	GT + GG vs. TT	1.655 (1.115–2.455)	**0.012**	584.139
Recessive	GG vs. TT + GT	1.261 (0.631–2.521)	0.512	590.016
Overdominant	GT vs. GG + TT	1.570 (1.053–2.342)	0.027	585.575
Additive	T	1.403 (1.040–1.892)	0.026	585.560
*STAT4* rs7601754				
Co-dominant	AG vs. GG AA vs. GG	0.577 (0.338–0.986) 0.659 (0.135–3.222)	0.044 0.607	587.939
Dominant	AG + AA vs. GG	0.584 (0.348–0.977)	0.041	585.964
Recessive	AA vs. GG + AG	0.730 (0.150–3.557)	0.697	590.276
Overdominant	AG vs. AA + GG	0.582 (0.341–0.994)	0.047	586.223
Additive	G	0.632 (0.396–1.008)	0.054	586.423
*STAT4* rs10168266				
Co-dominant	CT vs. TT CC vs. TT	1.386 (0.910–2.110) 1.808 (0.723–4.520)	0.128 0.205	587.104
Dominant	CT + CC vs. TT	1.432 (0.957–2.142)	0.080	587.405
Recessive	CC vs. TT + AT	1.616 (0.655 -3.988)	0.298	589.396
Overdominant	CT vs. CC + TT	0.863 (0.559–1.333)	0.177	588.632
Additive	T	1.367 (0.979–1.909)	0.066	587.115

PA—pituitary adenoma; OR—odds ratio; AIC—Akaike information criterion; *p*-value—significance level. Bonferroni correction applied to the significance level when *p* < 0.0125 (0.05/4). Statistically significant results are marked in bold. The most robust genetic model is underlined (selected based on the lowest AIC value).

**Table 4:** Distribution of genotypes and alleles of *STAT4* rs10181656, rs7574865, rs7601754, and rs10168266 polymorphisms within PA and reference group males.

Polymorphism	PA, N (%)	Reference Group, N (%)	*p*-Value
*STAT4* rs10181656			
CC	27 (47.4)	83 (61.0)	0.215
CG	24 (42.1)	43 (31.6)	
GG	6 (10.5)	10 (7.4)	
Total	57 (100)	136 (100)	
Allele			
C	78 (68.4)	209 (76.8)	0.084
G	36 (31.6)	63 (23.2)	
*STAT4* rs7574865			
GG	26 (45.6)	85 (62.5)	0.091
GT	24 (42.1)	41 (30.1)	
TT	7 (12.3)	10 (7.4)	
Total	57 (100)	136 (100)	
Allele			
G	76 (66.7)	211 (77.6)	0.033
T	38 (33.3)	61 (22.4)	
*STAT4* rs7601754			
AA	48 (84.2)	105 (77.2)	0.450
AG	7 (12.3)	27 (19.9)	
GG	2 (3.5)	4 (2.9)	
Total	57 (100)	136 (100)	
Allele			
A	103 (90.4)	237 (87.1)	0.373
G	11 (9.6)	35 (12.9)	
*STAT4* rs10168266			
CC	29 (50.9) ^1^	98 (72.1) ^1^	0.016
CT	24 (42.1)	34 (25.0)	
TT	4 (7.0)	4 (2.9)	
Total	57 (100)	136 (100)	
Allele			
C	82 (71.9)	230 (84.6)	**0.004**
T	32 (28.1)	42 (15.4)	

^1^ *p* = 0.005 (CC vs. CT + TT); *p*-value—significance level. Bonferroni correction applied to the significance level when *p* < 0.0125 (0.05/4). Statistically significant results are in bold.

**Table 5:** Binary logistic regression analysis of *STAT4* rs10181656, rs7574865, rs7601754, and rs10168266 within males with pituitary adenoma and reference group males.

Model	Genotype/Allele	OR (95% CI)	*p*-Value	AIC
*STAT4* rs10181656				
Co-dominant	CG vs. GG CC vs. GG	1.716 (0.885–3.326) 1.844 (0.613–5.548)	0.110 0.276	235.192
Dominant	CG + CC vs. GG	1.740 (0.933–3.247)	0.082	233.208
Recessive	CC vs. GG + CG	1.482 (0.512–4.292)	0.468	235.738
Overdominant	CG vs. CC + GG	1.573 (0.831–2.977)	0.164	234.329
Additive	G	1.481 (0.927–2.366)	0.100	233.573
*STAT4* rs7574865				
Co-dominant	GT vs. TT GG vs. TT	1.914 (0.981–3.734) 2.288 (0.792–6.612)	0.057 0.126	231.490
Dominant	GT + GG vs. TT	1.987 (1.062–3.717)	0.032	231.593
Recessive	GG vs. TT + GT	1.764 (0.636–4.892)	0.275	235.100
Overdominant	GT vs. GG + TT	1.685 (0.888–3.198)	0.110	233.724
Additive	T	1.639 (1.032–2.603)	0.036	231.886
*STAT4* rs7601754				
Co-dominant	AG vs. GG AA vs. GG	0.567 (0.231–1.393) 1.094 (0.194–6.178)	0.216 0.919	236.560
Dominant	AG + AA vs. GG	0.635 (0.281–1.438)	0.276	235.000
Recessive	AA vs. GG + AG	1.200 (0.214–6.744)	0.836	236.206
Overdominant	AG vs. AA + GG	0.565 (0.231–1.385)	0.212	234.570
Additive	G	0.755 (0.386–1.476)	0.411	235.537
*STAT4* rs10168266				
Co-dominant	CT vs. TT CC vs. TT	2.385 (1.224–4.647) 3.379 (0.795–14.356)	**0.011** 0.099	230.229
Dominant	CT + CC vs. TT	2.490 (1.313–4.724)	**0.005**	228.441
Recessive	CC vs. TT + AT	2.491 (0.601–10.325)	0.209	234.711
Overdominant	CT vs. CC + TT	2.182 (1.135–4.194)	0.019	230.833
Additive	T	2.122 (1.244–3.620)	**0.006**	228.556

PA—pituitary adenoma; OR—odds ratio; AIC—Akaike information criterion; *p*-value—significance level. Bonferroni correction applied to the significance level when *p* < 0.0125 (0.05/4). Statistically significant results marked in bold. The most robust genetic model is underlined (selected based on the lowest AIC value).

**Table 6:** Distribution of genotypes and alleles of *STAT4* rs10181656, rs7574865, rs7601754, and rs10168266 polymorphisms within micro or macro pituitary adenoma and reference groups.

Polymorphism	Reference Group, *n* (%)	Micro PA, *n* (%)	Macro PA, *n* (%)	*p*-Value
*STAT4* rs10181656				
CC	208 (58.3)	26 (52.0)	39 (43.8)	0.575 *
CG	123 (34.5)	21 (42.0)	41 (46.1)	0.049 **
GG	26 (7.3)	3 (6.0)	9 (10.1)	
Total	357 (100)	50 (100)	89 (100)	
Allele				
C	539 (75.5)	73 (73.0)	119 (66.9)	0.589 *
G	175 (25.5)	27 (37.0)	59 (33.1)	0.019 **
*STAT4* rs7574865				
GG	209 (58.5) ^1^	25 (50.0)	39 (43.8) ^1^	0.372 *
GT	121 (33.9)	22 (44.0)	40 (44.9)	0.042 **
TT	27 (7.6)	3 (6.0)	10 (11.2)	
Total	357 (100)	50 (100)	89 (100)	
Allele				
G	539 (75.5)	72 (72.0)	118 (66.3)	0.450 *
T	175 (25.5)	28 (28.0)	60 (33.7)	0.013 **
*STAT4* rs7601754				
AA	270 (75.6)	40 (80.0)	77 (86.5)	0.413 *
AG	80 (22.4)	8 (16.0)	12 (13.5)	0.061 **
GG	7 (2.0)	2 (4.0)	0 (0.0)	
Total	357 (100)	50 (100)	89 (100)	
Allele				
A	620 (86.8)	88 (88.0)	166 (93.3)	0.745 *
G	94 (13.2)	12 (12.0)	12 (6.7)	0.018 **
*STAT4* rs10168266				
CC	238 (66.7)	27 (54.0)	54 (60.7)	0.199 *
CT	106 (29.7)	21 (42.0)	29 (32.6)	0.334 **
TT	13 (3.6)	2 (4.0)	6 (6.7)	
Total	357 (100)	50 (100)	89 (100)	
Allele				
C	582 (81.5)	75 (75.0)	137 (77.0)	0.122 *
T	132 (18.5)	25 (25.0)	41 (23.0)	0.170 **

^1^ *p* value (GG vs. GT + TT) = 0.012; * micro PA vs. reference group; ** macro PA vs. reference group.

**Table 7:** Distribution of genotypes and alleles of *STAT4* rs10181656, rs7574865, rs7601754, and rs10168266 polymorphisms within pituitary adenoma groups with or without relapse and reference groups.

Polymorphism	Reference Group, *n* (%)	PA Without Relapse, *n* (%)	PA with Relapse, *n* (%)	*p*-Value
*STAT4* rs10181656				
CC	208 (58.3)	48 (44.9)	17 (53.8)	0.050 *
CG	123 (34.5)	49 (45.8)	13 (40.6)	0.780 **
GG	26 (7.3)	10 (9.3	2 (6.3)	
Total	357 (100)	107 (100)	32 (100)	
Allele				
C	539 (75.5)	145 (67.8)	47 (73.4)	0.024 *
G	175 (25.5)	69 (32.2)	17 (26.6)	0.715 **
*STAT4* rs7574865				
GG	209 (58.5)	47 (43.9)	17 (53.8)	0.029 *
GT	121 (33.9)	49 (45.8)	13 (40.6)	0.740 **
TT	27 (7.6)	11 (10.3)	2 (6.3)	
Total	357 (100)	107 (100)	32 (100)	
Allele				
G	539 (75.5)	143 (66.8)	47 (73.4)	0.012 *
T	175 (25.5)	71 (33.2)	17 (26.6)	0.715 **
*STAT4* rs7601754				
AA	270 (75.6)	89 (83.2)	28 (87.5)	0.244 *
AG	80 (22.4)	16 (15.0)	4 (12.5)	0.286 **
GG	7 (2.0)	2 (1.9)	0 (0.0)	
Total	357 (100)	107 (100)	32 (100)	
Allele				
A	620 (86.8)	194 (90.7)	60 (93.8)	0.135 *
G	94 (13.2)	20 (9.3)	4 (6.2)	0.110 **
*STAT4* rs10168266				
CC	238 (66.7)	61 (57.0)	20 (62.5)	0.135 *
CT	106 (29.7)	39 (36.4)	11 (34.4)	0.855 **
TT	13 (3.6)	7 (6.5)	1 (3.1)	
Total	357 (100)	107 (100)	32 (100)	
Allele				
C	582 (81.5)	161 (75.2)	51 (79.7)	0.044 *
T	132 (18.5)	53 (24.8)	13 (20.3)	0.719 **

* PA without relapse vs. reference group; ** PA with relapse vs. reference group.

**Table 8:** Binary logistic regression analysis within PA with or without relapse and reference group subjects.

Model	Genotype/Allele	OR (95% CI)	*p*-Value	AIC
PA with relapse				
*STAT4* rs10181656				
Co-dominant	CG vs. GG CC vs. GG	1.293 (0.607–2.753) 0.941 (0.206–4.307)	0.505 0.938	224.666
Dominant	CG + CC vs. GG	1.232 (0.596–2.544)	0.573	222.839
Recessive	CC vs. GG + CG	0.849 (0.192–3.751)	0.829	223.106
Overdominant	CG vs. CC + GG	1.302 (0.622–2.724)	0.484	222.672
Additive	G	1.107 (0.630–1.945)	0.723	223.031
*STAT4* rs7574865				
Co-dominant	GT vs. TT GG vs. TT	1.321 (0.620–2.813) 0.911 (0.199–4.160)	0.471 0.904	224.563
Dominant	GT + GG vs. TT	1.246 (0.603–2.574)	0.552	222.803
Recessive	GG vs. TT + GT	0.815 (0.185–3.594)	0.787	223.077
Overdominant	GT vs. GG + TT	1.334 (0.638–2793)	0.444	222.578
Additive	T	1.106 (0.632–1.935)	0.725	223.032
*STAT4* rs7601754				
Co-dominant	AG vs. GG AA vs. GG	0.482 (0.164–1.415) -	0.184 -	221.879
Dominant	AG + AA vs. GG	0.443 (0.151–1.299)	0.138	220.534
Recessive	AA vs. GG + AG	-	-	-
Overdominant	AG vs. AA + GG	0.495 (0.169–1.452)	0.200	221.244
Additive	G	0.444 (0.158–1.247)	0.123	220.187
*STAT4* rs10168266				
Co-dominant	CT vs. TT CC vs. TT	1.235 (0.571–2.668) 0.915 (0.114–7.360)	0.591 0.934	224.848
Dominant	CT + CC vs. TT	1.200 (0.568–2.537)	0.633	222.930
Recessive	CC vs. TT + AT	0.854 (0.108–6.744)	0.881	223.131
Overdominant	CT vs. CC + TT	1.240 (0.578–2.663)	0.581	222.855
Additive	T	1.123 (0.595–2.119)	0.721	223.029
PA without relapse				
*STAT4* rs10181656				
Co-dominant	CG vs. GG CC vs. GG	1.726(1.094–2.724) 1.667 (0.753–3.687)	0.019 0.207	499.163
Dominant	CG + CC vs. GG	1.716 (1.110–2.651)	0.015	497.170
Recessive	CC vs. GG + CG	1.312 (0.612–2.817)	0.485	502.654
Overdominant	CG vs. CC + GG	1.607 (1.037–2.492)	0.034	498.665
Additive	G	1.444 (1.040–2.006)	0.028	498.392
*STAT4* rs7574865				
Co-dominant	GT vs. TT GG vs. TT	1.801 (1.138–2.848) 1.812 (0.840–3.909)	**0.012** 0.130	498.041
Dominant	GT + GG vs. TT	1.803 (1.166–2.788)	**0.008**	496.041
Recessive	GG vs. TT + GT	1.400 (0.670–2.926)	0.370	502.354
Overdominant	GT vs. GG + TT	1.648 (1.062 -2.556)	0.026	498.197
Additive	T	1.495 (1.081–2.069)	0.015	497.314
*STAT4* rs7601754				
Co-dominant	AG vs. GG AA vs. GG	0.607 (0.337–1.092) 0.860 ((0.177–4.249)	0.096 0.860	502.143
Dominant	AG + AA vs. GG	0.628 (0.358–1.100)	0.104	500.311
Recessive	AA vs. GG + AG	0.952 (0.195–4.654)	0.952	503.120
Overdominant	AG vs. AA + GG	0.609 (0.339–1.095)	0.097	500.175
Additive	G	0.689 (0.417–1.138)	0.146	500.854
*STAT4* rs10168266				
Co-dominant	CT vs. TT CC vs. TT	1.436 (0.904–2.280) 2.101 (0.804–5.492)	0.125 0.130	501.282
Dominant	CT + CC vs. TT	1.508 (0.970–2.345)	0.068	499.833
Recessive	CC vs. TT + AT	1.852 (0.720–4.768)	0.201	501.595
Overdominant	CT vs. CC + TT	1.358 (0.862–2.139)	0.187	501.407
Additive	T	1.442 (1.004–2.071)	0.047	499.283

PA—pituitary adenoma; OR—odds ratio; AIC—Akaike information criterion; *p*-value—significance level. Bonferroni correction applied at the significance level when *p* < 0.0125 (0.05/4). Statistically significant results marked in bold. The most robust genetic model is underlined (selected based on the lowest AIC value).

## Data Availability

The datasets used and analyzed during the current study are available from the corresponding author upon reasonable request.

## Supplementary Materials

The following supporting information can be downloaded at: https://www.mdpi.com/article/10.3390/medicina60111871/s1, Table S1. Distribution of genotypes and alleles of STAT4 rs10181656, rs7574865, rs7601754, and rs10168266 polymorphisms in females within PA and reference groups females; Table S2. Binary logistic regression analysis of STAT4 rs10181656, rs7574865, rs7601754, and rs10168266 within females with pituitary adenoma and reference group females; Table S3. Binary logistic regression analysis within micro or macro PA and reference group subjects; Table S4. Linkage disequilibrium between studied polymorphisms in patients with PA; Table S5. Haplotype association of STAT4 rs10181656, rs7574865, rs7601754, and rs10168266 with the predisposition to PA occurrence.
