# Supplementary material for: Signal Transducer and Activator of Transcription 4 (STAT4) Association with Pituitary Adenoma
Source: Medicina (Kaunas). 2024 Nov 14;60(11):1871. doi: 10.3390/medicina60111871 (PMC11596619; doi:10.3390/medicina60111871)
Supplement: Supplementary file 1 [file medicina-60-01871-s001.zip › medicina-3250186-supplementary.pdf]

Supplementary material

Table S1. Distribution of genotypes and alleles of *STAT4* rs10181656, rs7574865, rs7601754, and rs10168266 polymorphisms in females within PA and reference groups females.

Polymorphism	PA, N (%)	Reference group, N (%)	p-value
STAT4 rs10181656			
CC	38 (46.3)	125 (56.6)	0.254
CG	38 (46.3)	80 (36.2)	
GG	6 (7.3)	16 (7.2)	
Total	82 (100)	221 (100)	
Allele			
C	114 (69.5)	330 (74.7)	0.203
G	50 (30.5)	112 (25.3)	
STAT4 rs7574865			
GG	38 (46.3)	124 (56.1)	0.265
GT	38 (46.3)	80 (36.2)	
TT	6 (7.3)	17 (7.7)	
Total	82 (100)	221 (100)	
Allele			
G	114 (69.5)	328 (74.2)	0.248
T	50 (30.5)	114 (25.8)	
STAT4 rs7601754			
AA	69 (84.1)	165 (74.7)	0.163
AG	13 (15.9)	53 (24.0)	
GG	0 (0.0)	3 (1.4)	
Total	82 (100)	221 (100)	
Allele			
A	151 (92.1)	383 (86.7)	0.067
G	13 (7.9)	59 (13.3)	
STAT4			
rs10168266 CC	52 (63.4)	140 (63.3)	0.949
CT	26 (31.7)	72 (32.6)	
TT	4 (4.9)	9 (4.1)	
Total	82 (100)	221 (100)	
Allele			
C	130 (79.3)	352 (79.6)	0.920
T	34 (20.7)	90 (20.4)	

p-value – significance level. Bonferroni corrected the significance level when p < 0.0125 (0.05/4).

Table S2. Binary logistic regression analysis of *STAT4* rs10181656, rs7574865, rs7601754, and rs10168266 within females with pituitary adenoma and reference group females.

Model	Genotype/allele	OR (95% CI)	p-value	AIC
		STAT4 rs10181656		
Co-dominant	CG vs. GG	1.562 (0.920-2.655)	0.099	355.110
	CC vs. GG	1.234 (0.451-3.374)	0.683	
Dominant	CG+CC vs. GG	1.508 (0.906-2.508)	0.114	353.324
Recessive	CC vs. GG+CG	1.012 (0.382-2.680)	0.982	355.832
Overdominant	CG vs. CC+GG	1.522 (0.911-2.544)	0.109	353.273
Additive	G	1.291 (0.869-1.919)	0.206	354.248
		STAT4 rs7574865		

Co-dominant	GT vs. TT	1.550 (0.912-2.634)	0.105	355.198
	GG vs. TT	1.152 (0.424-3.128)	0.782	
Dominant	GT+GG vs. TT	1.480 (0.890-2.562)	0.131	353.543
Recessive	GG vs. TT+GT	0.947 (0.360-2.492)	0.913	355.820
Overdominant	GT vs. GG+TT	1.522 (0.911-2.544)	0.109	353.273
Additive	T	1.259 (0.849-1.865)	0.252	354.533
STAT4 rs7601754				
Co-dominant	AG vs. GG	0.587 (0.301-1.145)	0.118	353.316
	AA vs. GG	-	-	
Dominant	AG+AA vs. GG	0.555 (0.285-1.080)	0.083	352.600
Recessive	AA vs. GG+AG	-	-	-
Overdominant	AG vs. AA+GG	0.597 (0.306-1.165)	0.131	353.397
Additive	G	0.546 (0.287-1.037)	0.065	352.071
STAT4 rs10168266				
Co-dominant	CT vs. TT	0.972 (0.561-1.685)	0.920	357.730
	CC vs. TT	1.197 (0.353-4.053)	0.773	
Dominant	CT+CC vs. TT	0.997 (0.589-1.688)	0.992	355.832
Recessive	CC vs. TT+AT	1.208 (0.362-4.035)	0.759	355.740
Overdominant	CT vs. CC+TT	0.961 (0.558-1.655)	0.885	355.811
Additive	T	1.023 (0.658-1.590)	0.920	355.822

PA – Pituitary Adenoma; OR – odds ratio; AIC – Akaike information criterion; p-value – significance level. Bonferroni corrected the significance level when p < 0.0125 (0.05/4); Statistically significant results marked in bold; The most robust genetic model underlined (selected based on the lowest AIC value).

Table S3. Binary logistic regression analysis within micro or macro PA and reference group subjects.

**Model**	**Genotype/allele**	**OR (95% CI)**	**p-value**	**AIC**
Micro PA				
STAT4 rs10181656				
Co-dominant	CG vs. GG	1.366 (0.737-2.531)	0.322	306.183
	CC vs. GG	0.923 (0.261-3.263)	0.901	
Dominant	CG+CC vs. GG	1.289 (0.712-2.332)	0.402	304.570
Recessive	CC vs. GG+CG	0.813 (0.237-2.790)	0.742	305.154
Overdominant	CG vs. CC+GG	1.378 (0.754-2.517)	0.297	304.199
Additive	G	1.132 (0.714-1.795)	0.599	304.996
STAT4 rs7574865				
Co-dominant	GT vs. TT	1.520 (0.822-2.812)	0.182	305.342
	GG vs. TT	0.929 (0.263-3.284)	0.909	
Dominant	GT+GG vs. TT	1.412 (0.780-2.555)	0.254	303.971
Recessive	GG vs. TT+GT	0.780 (0.228-2.673)	0.693	305.103
Overdominant	GT vs. GG+TT	1.532 (0.841-2.792)	0.163	303.355
Additive	T	1.185 (0.752-1.867)	0.464	304.743
STAT4 rs7601754				
Co-dominant	AG vs. GG	0.675 (0.304-1.501)	0.335	305.567
	AA vs. GG	1.929 (0.387-9.611)	0.423	
Dominant	AG+AA vs. GG	0.776 (0.372-1.616)	0.498	304.791
Recessive	AA vs. GG+AG	2.083 (0.421-10.320)	0.369	304.562
Overdominant	AG vs. AA+GG	0.660 (0.298-1.462)	0.305	304.138
Additive	G	0.903 (0.482-1.692)	0.751	305.166
STAT4 rs10168266				
Co-dominant	CT vs. TT	1.746 (0.945-3.228)	0.075	304.165
	CC vs. TT	1.356 (0.290-6.332)	0.698	
Dominant	CT+CC vs. TT	1.704 (0.937-3.098)	0.081	302.272
Recessive	CC vs. TT+AT	1.103 (0.241-5.036)	0.900	305.253
Overdominant	CT vs. CC+TT	1.715 (0.936-3.142)	0.081	302.306

Additive	T	1.473 (0.899-2.413)	0.124	303.003
Macro PA				
STAT4 rs10181656				
Co-dominant	CG vs. GG	1.778 (1.087-2.907)	0.022	443.813
	CC vs. GG	1.846 (0.804-4.241)	0.148	
Dominant	CG+CC vs. GG	1.790 (1.120-2.859)	0.015	441.821
Recessive	CC vs. GG+CG	1.432 (0.646-3.176)	0.377	447.060
Overdominant	CG vs. CC+GG	1.625 (1.015-2.601)	0.043	443.756
Additive	G	1.499 (1.057-2.126)	0.023	442.756
STAT4 rs7574865				
Co-dominant	GT vs. TT	1.772 (1.080-2.905)	0.023	443.509
	GG vs. TT	1.985 (0.890-4.426)	0.094	
Dominant	GT+GG vs. TT	1.810 (1.133-2.893)	0.013	441.584
Recessive	GG vs. TT+GT	1.547 (0.719-3.328)	0.264	446.626
Overdominant	GT vs. GG+TT	1.592 (0.993-2.552)	0.053	444.112
Additive	T	1.523 (1.078-2.150)	0.017	442.212
STAT4 rs7601754				
Co-dominant	AG vs. GG	0.526 (0.273-1.015)	0.055	442.585
	AA vs. GG	-	-	
Dominant	AG+AA vs. GG	0.484 (0.251-0.931)	0.030	442.465
Recessive	AA vs. GG+AG	-	-	-
Overdominant	AG vs. AA+GG	0.540 (0.280-1.041)	0.066	444.058
Additive	G	0.479 (0.256-0.896)	0.021	441.557
STAT4 rs10168266				
Co-dominant	CT vs. TT	1.206 (0.727-2.000)	0.468	447.784
	CC vs. TT	2.034 (0.740-5.593)	0.169	
Dominant	CT+CC vs. TT	1.296 (0.803-2.093)	0.288	446.689
Recessive	CC vs. TT+AT	1.913 (0.706-5.182)	0.202	446.303
Overdominant	CT vs. CC+TT	1.144 (0.696-1.883)	0.595	447.524
Additive	T	1.309 (0.885-1.935)	0.178	446.037

PA – Pituitary Adenoma; OR – odds ratio; AIC – Akaike information criterion; p-value – significance level. Bonferroni corrected the significance level when p < 0.0125 (0.05/4); Statistically significant results marked in bold; The most robust genetic model underlined (selected based on the lowest AIC value).

Table S4. Linkage disequilibrium between studied polymorphisms in patients with PA.

SNP-SNP	D'	r2	p-value
rs10181656-rs7574865	0.979	0.949	< 0.001
rs10181656-rs7601754	0.918	0.041	< 0.001
rs10181656-rs10168266	0.796	0.443	< 0.001
rs7574865-rs7601754	0.920	0.041	< 0.001
rs7574865-rs10168266	0.827	0.473	< 0.001
rs7601754-rs10168266	0.863	0.025	< 0.001

SNP – single nucleotide polymorphism; D': the deviation between the expected haplotype frequency and the observed frequency; r^2: the square of the haplotype frequency correlation coefficient; p-value – significance level when p < 0.05.

Table S5. Haplotype association of *STAT4* rs10181656, rs7574865, rs7601754, and rs10168266 with the predisposition to PA occurrence.

**Haplotype No.**	***STAT4* rs10181656**	***STAT4* rs7574865**	***STAT4* rs7601754**	***STAT4* rs10168266**	**Frequency**		**OR (95% CI)**	**p-value**
					**PA**	**Reference**		
1	C	G	A	C	56.87	59.73	1	NA
2	G	T	A	T	19.82	15.92	1.310 (0.900-1.890)	0.160
3	C	G	G	C	8.27	12.91	0.670 (0.410-1.080)	0.100
4	G	T	A	C	10.75	8.17	1.370 (0.850-2.220)	0.200
5	C	G	A	T	2.84	2.31	1.270 (0.500-3.230)	0.610

OR: odds ratio; CI: confidence interval; p-value: significance level (statistically significant when $p < 0.05/4$).
